# Supplementary figures and images for: Germinal center trajectories and transcriptional signatures define CLL subtypes and their pathway regulators
Source: PLoS One. 2025 Oct 22;20(10):e0335069. doi: 10.1371/journal.pone.0335069 (PMC12543202; doi:10.1371/journal.pone.0335069)

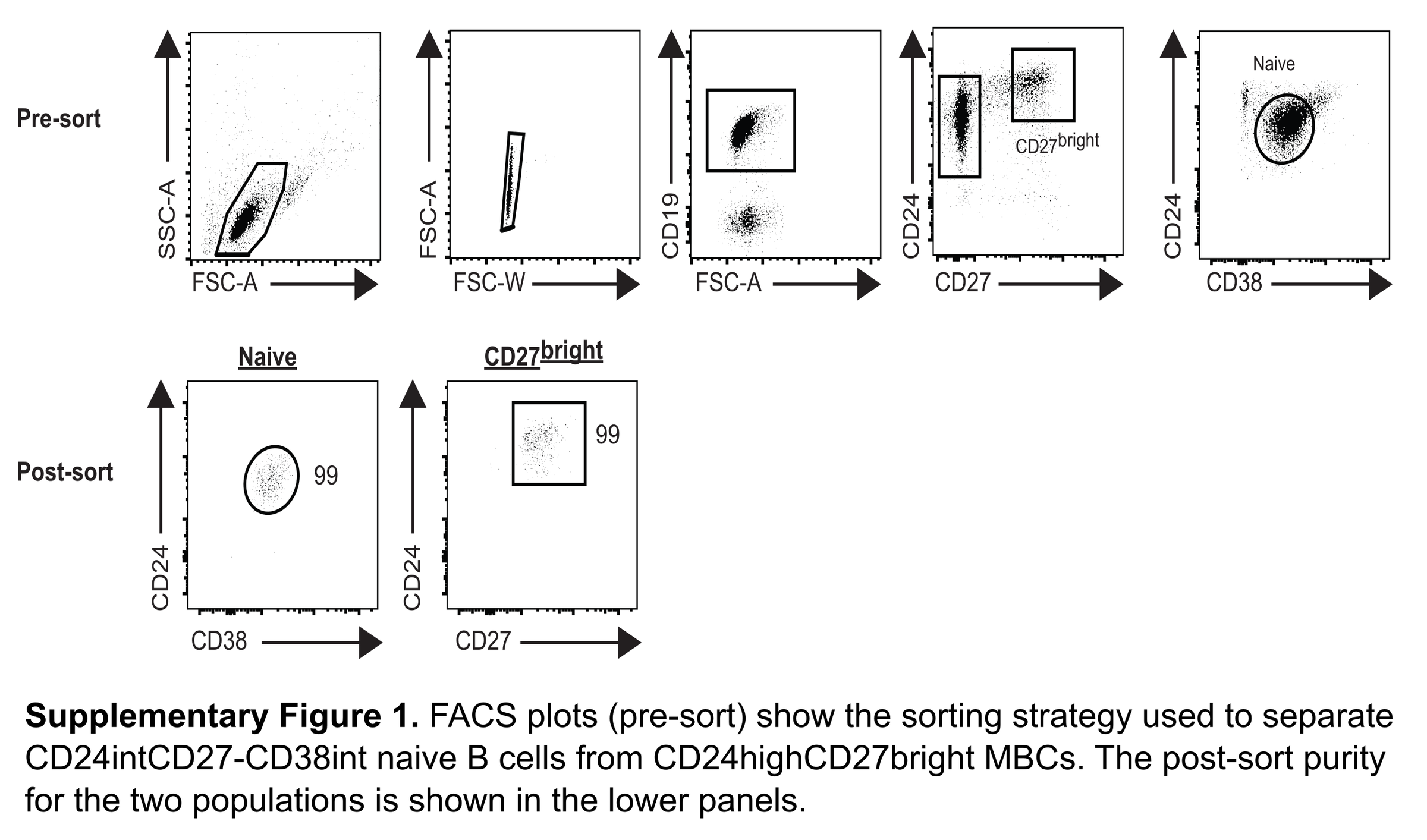

Supplement: S1 Fig — FACS plots (pre-sort) show the sorting strategy used to separate CD24intCD27-CD38int naive B cells from CD24highCD27bright MBCs. The post-sort purity for the two populations is shown in the lower panels. (TIF) [file pone.0335069.s001.tif]

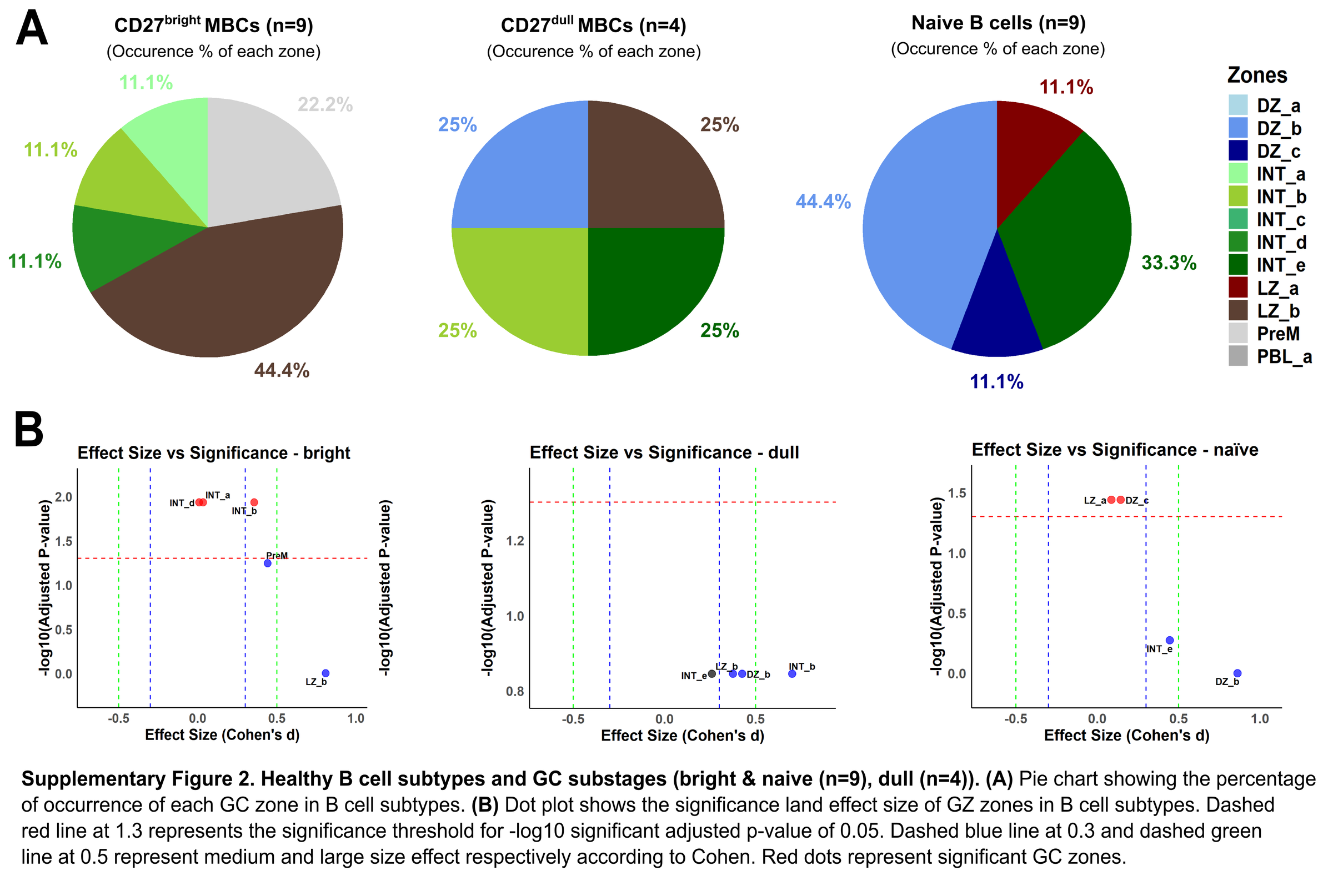

Supplement: S2 Fig — (A) Pie chart showing the percentage of occurrence of each GC zone in B cell subtypes. (B) Dot plot shows the significance land effect size of GZ zones in B cell subtypes. Dashed red line at 1.3 represents the significance threshold for -log10 significant adjusted p-value of 0.05. Dashed blue line at 0.3 and dashed green line at 0.5 represent medium and large size effect respectively according to Cohen. Red dots represent significant GC zones. (TIF) [file pone.0335069.s002.tif]

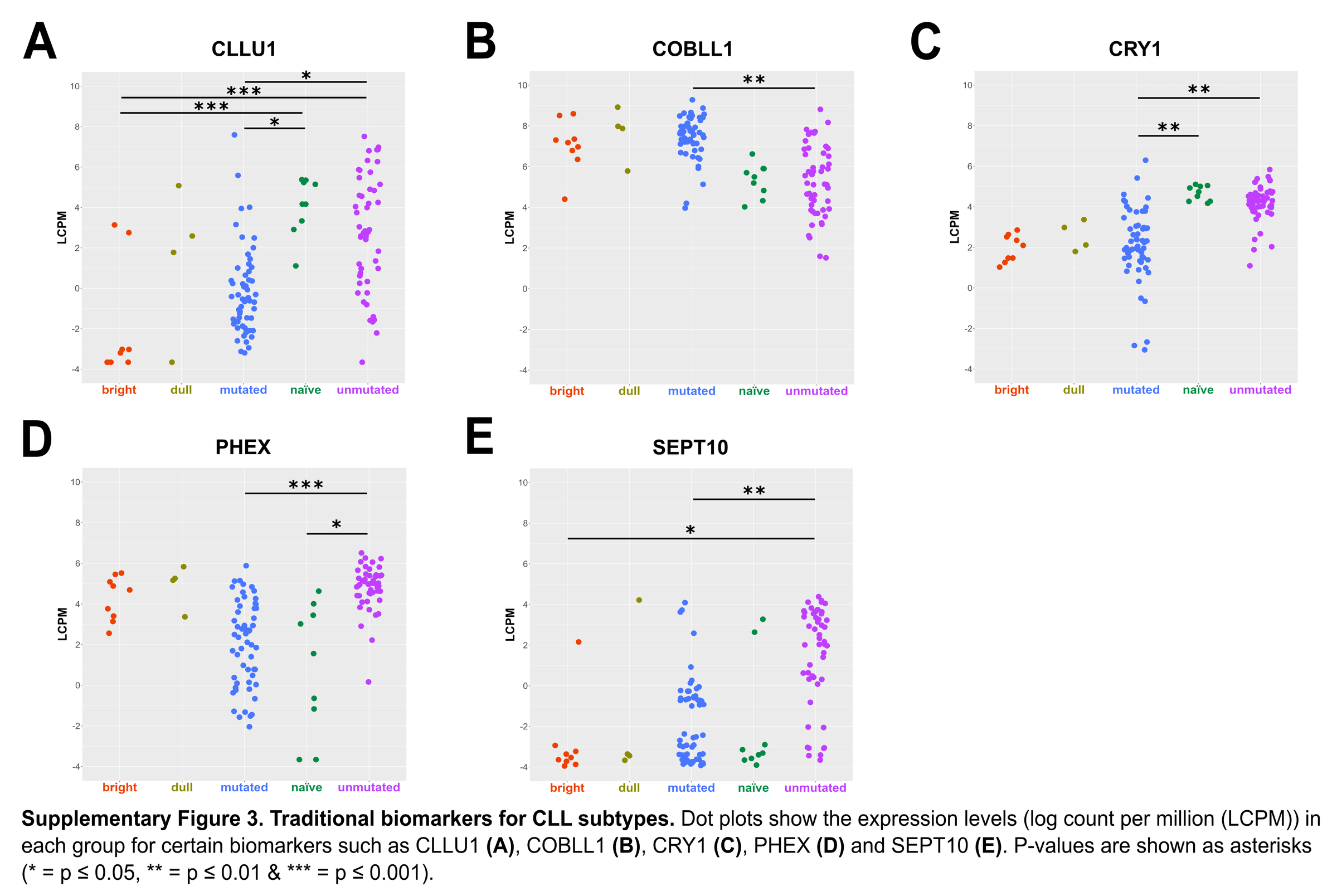

Supplement: S3 Fig — Dot plots show the expression levels (log count per million (LCPM)) in each group for certain biomarkers such as CLLU1 (A), COBLL1 (B), CRY1 (C), PHEX (D) and SEPT10 (E). P-values are shown as asterisks (* = p ≤ 0.05, ** = p ≤ 0.01 & *** = p ≤ 0.001). (TIF) [file pone.0335069.s003.tif]
