# Supplementary material for: Germinal center trajectories and transcriptional signatures define CLL subtypes and their pathway regulators
Source: PLoS One. 2025 Oct 22;20(10):e0335069. doi: 10.1371/journal.pone.0335069 (PMC12543202; doi:10.1371/journal.pone.0335069)
Supplement: Attachment — Submitted filename: Response to reviewers.docx [file pone.0335069.s012.docx]

Dear reviewers,

We deeply regret your decision to reject our submission (PONE-D-25-12115) titled *Germinal center trajectories and transcriptional signatures define CLL subtypes and their functional regulators* because of ethical concerns. We have tried to make clear that there are no ethical concerns and that we have followed the laws of the country of origin of the samples (Sweden). We have produced the data on healthy donors from anonymous buffy coats in Sweden in our laboratory, thus this is in house data from a different project of ours for which we have a valid ethical permit that we sent to you. We apologize for not making this even more clear earlier on.

We have now added, to the manuscript body, the date of access to the data as well as the period of recruitment to the other study where we have the valid ethical permit to recruit healthy donors from anonymous buffy coats. We have also updated the Human Participants Checklist, the ethical permit and translation of it. We have also removed the figures from the manuscript body and updated the cover letter.

We apologize for uploading the wrong and old version of the manuscript by mistake during the last adjustments in the previous submission. We hope you understand that it was only a confusion with the uploading process.

With all this in mind we hope that you will be able to re-consider our manuscript as a new submission to PLOS ONE. We appreciate your time and consideration of our manuscript and look forward to your response. Please do not hesitate to contact us if further information is needed.

Yours sincerely,

*Ahmed Mohamed, MSc*
