# Supplementary material for: Germinal center trajectories and transcriptional signatures define CLL subtypes and their pathway regulators
Source: PLoS One. 2025 Oct 22;20(10):e0335069. doi: 10.1371/journal.pone.0335069 (PMC12543202; doi:10.1371/journal.pone.0335069)
Supplement: Attachment — Submitted filename: Response to reviewers_revision.docx [file pone.0335069.s013.docx]

Dear reviewers and editor,

Thank you for reviewing our manuscript and providing constructive feedback. We adjusted the manuscript according to your feedback and we added here more detailed answers to your concerns.

**Editor:**

“Please ensure that your manuscript meets PLOS ONE's style requirements, including those for file naming.”

The style of the manuscript and file naming have been adjusted to meet PLOS ONE requirements. Figures have been uploaded to PACE to ensure the compatibility and uploaded again with this revision.

“Please include a complete copy of PLOS’ questionnaire on inclusivity in global research in your revised manuscript.”

A complete PLOS’ questionnaire on inclusivity in global research has been uploaded in the “Supporting Information” category with this revision.

“Please state what role the funders took in the study.”

The role of funders has been amended in the cover letter.

“We note that you have indicated that there are restrictions to data sharing for this study. PLOS only allows data to be available upon request if there are legal or ethical restrictions on sharing data publicly.”

We have the data and analysis pipeline available open access on Zenodo (DOI: 10.5281/zenodo.15000801). Only the raw data (fastq files) of one cohort (second healthy dataset in the manuscript) is uploaded to NCBI with GSE307095, but not open access yet and it will be open access after the acceptance of this manuscript. We will not apply any restrictions.

“In the online submission form, you indicated that your data will be submitted to a repository upon acceptance.“

The data is already available open access on Zenodo with the previously mentioned DOI and no restrictions are applied.

“Your ethics statement should only appear in the Methods section of your manuscript. If your ethics statement is written in any section besides the Methods, please delete it from any other section.”

The ethics statement has been removed from all sections except methods.

“If the reviewer comments include a recommendation to cite specific previously published works, please review and evaluate these publications to determine whether they are relevant and should be cited. There is no requirement to cite these works unless the editor has indicated otherwise.”

No citations have been proposed.

“Please review your reference list to ensure that it is complete and correct. If you have cited papers that have been retracted, please include the rationale for doing so in the manuscript text, or remove these references and replace them with relevant current references. Any changes to the reference list should be mentioned in the rebuttal letter that accompanies your revised manuscript. If you need to cite a retracted article, indicate the article’s retracted status in the References list and also include a citation and full reference for the retraction notice.”

The reference list has been checked and no edits have been incorporated.

**Reviewer 1:**

“Tonsillar GC substages rely on niche-specific signals (e.g., CXCL12/CD40L) absent in circulation, risking misinterpretation of transcriptomic signatures as active GC maturation.”

*Response:* Thank you for pointing out that tonsillar GC substages relies on niche-specific signals (e.g., CXCL12, CD40L) that may not be present in circulation. We acknowledge the limitation of using these signatures in the CLL context and therefore we have added a sentence in the discussion on the limitation of using this dataset to map CLL samples to GC substages. We still find this approach valuable since the core transcriptional programs remain conserved for B cell states during GC maturation which allows potential mapping of CLL samples to GC substages. This dataset showed a comprehensive transcriptional resolution of GC substages in healthy humans.

“Protein-level validation (e.g., CXCR4/CD83 flow cytometry) and comparison to lymph node/bone marrow microenvironments are lacking.”

*Response:* We fully agree that protein-level validation and comparisons to lymph node or bone marrow niches would further strengthen our findings. Unfortunately, we have no access to lymph node samples to perform CXCR4/CD83 DZ vs LZ GC B cells stains. This would be one of the goals in a follow up study to compare our results to lymph node single cell RNA sequencing results from lymph node cells of CLL patients.

“Could the observed substage signatures reflect aberrant transcriptional mimicry (e.g., constitutive NF-κB) rather than true GC maturation? Please temper claims and discuss limitations. Additionally, please Revise Methods to passive voice for objectivity.”

*Response:* It is worth mentioning that this is a transcriptomics-focused study, but this approach can guide the direction of future work. We agree that some of the observed patterns, such as constitutive NF-κB activation, BCR signaling, or defective apoptotic regulation, could reflect aberrant transcriptional programs in CLL. In turn, it is rather a transcriptional similarity of CLL profiles to GC substages without implications that GC substages do occur in CLL. The results are not direct evidence of GC maturation in CLL, but existential discussion on what if CLL originates from GC. We have incorporated these limitations to the discussion section to clarify the interpretive boundaries of our findings. The methods section has been adjusted to passive voice for improved objectivity.

**Reviewer 2:**

“The manuscript title 'Germinal center trajectories and transcriptional signatures define CLL subtypes and their functional regulators,' suggests the inclusion of functional assays. However, based on the manuscript's content, the proposed pathways and gene signature relationships appear to be derived solely from in silico pathway analysis and the available literature. To accurately reflect the scope of the work, it is recommended to revise the title particularly regarding the 'functional regulators' aspect.

The same implies in the abstract also. The authors have mentioned that functional analysis revealed that LPL.....EMT regulation. The term functional analysis should be replaced with functional enrichment analysis. The abstract needs to clearly outline the methodology used in the manuscript. It needs to include details like number of patients studied, methodology like meta analysis was conducted.”

*Response:* Thank you for the insights into the phrasing context. We have modified the title and the abstract to avoid the term “functional analysis” which often refers to wet lab experiments and added the suggested term “enrichment”. We have also fixed the abstract to include the suggested details on the methodology and the description of the analysis.

“The transcriptional signatures highlighting the traditional and potential biomarkers for CLL sub- types is diverting the focus of the manuscript. Many published papers are already available for transcriptional signatures between these subtypes (Mutated vs unmutated). Hence, the abstract and discussion should be modified accordingly.”

*Response:* The second paragraph in the introduction has been shortened as well as the discussion part on the CLL transcriptional signatures.

“Given that the results are based on bioinformatics meta-analysis without functional validation, avoid making very strong conclusions. Please use mild words like 'suggested' or 'hypothesized' in both the abstract and discussion. A statement acknowledging that these findings require functional validation may be added somewhere in the discussion. The effort should be to make it concise. In the introduction section, paragraph 2 may be more concise. The discussion also needs to be modified and concise in line with the suggestions provided above.”

*Response:* Limitations of the study are listed now at the end of the discussion. The style of the text, in both abstract and discussion sections, was adjusted with modesty in the claims in consideration that it is transcriptomic data analysis and no validations have been carried out.

We appreciate your time and consideration of our manuscript and please do not hesitate to contact us if further information is needed.

Yours sincerely,

*Ahmed Mohamed, MSc*
